# Pyrolysis and Combustion of Polyvinyl Chloride (PVC) Sheath for New and Aged Cables via Thermogravimetric Analysis-Fourier Transform Infrared (TG-FTIR) and Calorimeter

**DOI:** 10.3390/ma11101997

**Published:** 2018-10-16

**Authors:** Zhi Wang, Ruichao Wei, Xuehui Wang, Junjiang He, Jian Wang

**Affiliations:** State Key Laboratory of Fire Science, University of Science and Technology of China, Hefei 230026, China; ustc14wz@mail.ustc.edu.cn (Z.W.); rcwei@mail.ustc.edu.cn (R.W.); wxuehui@mail.ustc.edu.cn (X.W.); hjj0513@mail.ustc.edu.cn (J.H.)

**Keywords:** aged cable, pyrolysis, TG-FTIR, combustion, calorimeter

## Abstract

To fill the shortages in the knowledge of the pyrolysis and combustion properties of new and aged polyvinyl chloride (PVC) sheaths, several experiments were performed by thermogravimetric analysis (TG), Fourier transform infrared (FTIR), microscale combustion calorimetry (MCC), and cone calorimetry. The results show that the onset temperature of pyrolysis for an aged sheath shifts to higher temperatures. The value of the main derivative thermogravimetric analysis (DTG) peak of an aged sheath is greater than that of a new one. The mass of the final remaining residue for an aged sheath is also greater than that of a new one. The gas that is released by an aged sheath is later but faster than that of a new one. The results also show that, when compared with a new sheath, the heat release rate (HRR) is lower for an aged one. The total heat release (THR) of aged sheath is reduced by 16.9–18.5% compared to a new one. In addition, the cone calorimetry experiments illustrate that the ignition occurrence of an aged sheath is later than that of a new one under different incident heat fluxes. This work indicates that an aged sheath generally pyrolyzes and it combusts more weakly and incompletely.

## 1. Introduction

Due to the outstanding electric insulation, prominent mechanical properties, high chemical resistance, natural flame retardant effect, ease of processing, efficient recyclability, etc., Polyvinyl chloride (PVC), one of the most extensively used plastics, has been widely applied in the wire and cable industry as the main constituent of insulation and sheathing [1,2,3]. However, PVC plastic that is commercially applied in cable sheathing is considerably flammable, even when treated by stabilizer, lubricant, plasticizer, and flame retardant [1]. The fire statistics illustrate that cable faults are among the most common causes of electrical fires, which involves the new and aged cables [4]. The aging degradation will lead to changes in initial properties as a result of simultaneous chemical and physical processes, causing changes in chemical composition and structure of materials [5]. There must be some differences of fire protection properties between the new and aged cables [2,4]. The outer PVC sheath is recognized as the main combustible part of a cable, and the investigation of its pyrolysis and combustion behavior is key to the study of fire properties of cables [4,6]. It will be aged firstly and fiercely by used for a long time. Limited systematic work has focused on comparing the pyrolysis and combustion properties between new and aged cable sheaths. Besides, as cables of the type used in this study are mostly used indoors, the temperature is considered as the most important factor to make them aged during the long-term service [2,5]. Thus, the thermal aging is supposed to simulate the natural indoors aging process.

Many studies have been conducted on the thermal degradation characteristics and combustion properties of typical PVC cable [7,8,9,10,11,12,13,14,15]. Benes et al. [16] used the thermogravimetric analysis (TG) coupled to mass spectrometry and Fourier transform infrared (FTIR) spectroscopy to study the thermal degradation of PVC cable under different atmospheres. It was proposed that the pyrolysis of PVC backbone is accompanied by the release of HCl, H_2_O, CO_2_ and benzene. Gao [17] explored the pyrolysis characteristics of insulative PVC materials applied for fire retardant cable. Wang et al. [10] performed several TG experiments coupled with FTIR analysis to determine the pyrolysis behavior of PVC sheath of flame-retarded cables. They proposed that the pyrolysis process for PVC sheath could be divided into two regions and the amount of six components were detected. Courty et al. [18] employed two tests method for the characterization of PVC/PVC cable pyrolysis and flammability. Fernandez-Pello et al. [19] studied the fire performance of seven types of complex cables, including PVC cable that is commonly used in electrical installations and focused on the ignition and flame spread. Andersson et al. [20] carried out both small and large scale fire experiments with PVC sheathed cable with PVC insulation around the individual wires under well-ventilated and vitiated conditions. McGrattan et al. [21,22] investigated the cable heat release, ignition, and spread in tray installations during a fire, corresponding to the PVC cable. Grayson et al. [23] studied the fire performance of several types of electric cables containing PVC cable to design improved standard testing methods to determine the fire property of cables. Matala et al. [1] investigated the effects of the modelling decisions and parameter estimation methods on the pyrolysis modelling of two PVC cables. It should be noted that all above works focus on the fresh PVC cables. Certainly, there are some studied on the aged PVC cable [2,3,4,5,24,25,26,27]. Quennehen et al. [24] analyzed the two sets of single core cables with PVC insulation to determine the aging mechanism that is responsible for this decrease of electrical properties. Jakubowicz et al. [5] studied the effects of accelerated and natural ageing on plasticized PVC cable and concluded that the accelerated ageing did not significantly affect the tensile properties of the insulation materials. Yu et al. [12] summarized thermal degradation of PVC waste. Emanuelsson et al. [2] studied the effect of accelerated aging on the fire performance of building wires involving one PVC-based cable and one flame-retarded polyolefin-based cable using cone calorimetry and FTIR. Wang et al. [28] performed experiments to estimate the fire characteristics of new and aged building wires using a cone calorimeter. Xie et al. [4] employed TG, FTIR, and microscale combustion calorimetry (MCC) to investigate the fire protection properties of PVC sheaths for new and old cables: the old one was taken from an old building’s electric power system, which had been in use for more than ten years; the new one represented a typical PVC cable manufactured at present. The new and old cables have significant differences in the compositions and structures due to the different commercial companies made at different time. Whereas, the current work is an integral study to compare the pyrolysis and combustion behaviors of the same cable sheath at different thermal aging degrees.

In this study, one flame-retardant PVC cable with different thermal aging degrees was adopted. The PVC sheath part removed from the cable was prepared to the follow-up tests. TG experiments were carried out to study the pyrolysis properties of PVC sheaths of new and aged cables with different heating rates (5, 10, 20, 30 and 40 K min^−1^) in nitrogen atmosphere. The onset temperatures of pyrolysis, mass loss, mass loss rate, and residue mass were recorded. Meanwhile, the gaseous release during the thermal degradation process was analyzed by TG coupled with FTIR spectroscopy. The combustion characteristics including heat release rate and total heat release were experimentally analyzed by MCC. In addition, a cone calorimeter was applied to investigate the time to ignition of new and aged cable sheaths. Finally, a comparison of the pyrolysis and combustion properties between new and aged cable sheaths was made and discussed.

## 2. Experimental

### 2.1. Sample Preparation

The sheath sample used in the present study was obtained from a flame-retardant PVC cable (ZR-RVV) that was provided by Jiangsu Xinchangfeng Cable Co., Ltd. in Wuxi, China. The main components of the sheath were PVC, antimony trioxide (Sb_2_O_3_), plasticizers, etc. Flame retardant accounts for about 7 wt %. The flame-retardant PVC cable was subjected to different degrees of thermal aging treatment to obtain the aged sheath sample. A rough elemental analysis of the sample was conducted, and the measured results can be observed in Table 1. It can be found that the contents of carbon and chlorine are decreased while the content of oxygen is increased with thermal aging degree. That could be ascribed to the dehydrochlorination and oxidation of the material during the thermal aging [24,29]. Varied sample preparation methods were applied to the tests. For the microscale TG and MCC experiment, the samples were milled to less than 0.5 mm. For the bench-scale cone calorimetry experiment, the cable was cut into pieces 10 cm long with 10 mm thickness. Prior to testing, all of the samples underwent a drying procedure at 80 °C for approximately 1 h to remove moisture.

### 2.2. Thermal Aging

**Figure 1:**
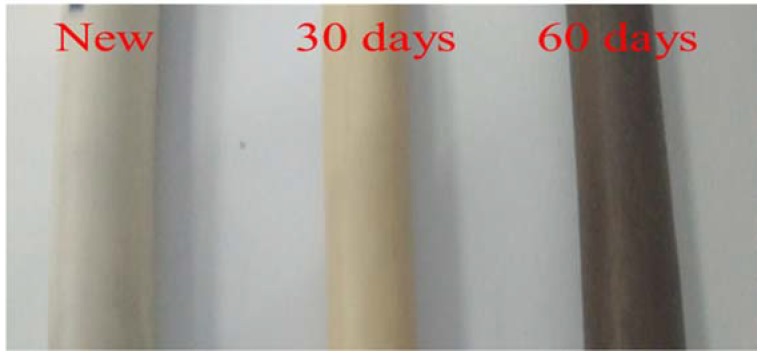
Digital photographs of polyvinyl chloride (PVC) sheaths before and after thermal aging.

Thermal aging of PVC sheaths was performed in a convection oven (GHX-100L, Hefei Anke Environmental Test Equipment Co., Ltd. in Hefei, China) in air at 100 ± 0.1 °C for 30 days and 60 days, respectively. After thermal aging, the samples presented a more pronounced brown color, as shown in Figure 1. Severe discoloration that occurred is due to the formation of chromophore groups [5]. The mass loss of PVC sheaths was monitored in the aging process using a precise balance, and the mass loss ratios were 10.2% for 30 days and 11.8% for 60 days when compared with the new sheath. Besides, the PVC sheath was also rigidized with prolonging the aging time. However, it is beyond the scope of the current study.

### 2.3. TG-FTIR Measurements

The tests were conducted by a TA Instruments SDT Q600 thermal analyzer (TA Instruments in New Castle, PA, USA) coupled with FTIR spectroscopy (PerkinElmer in Waltham, MA, USA). The TG test ranged from 303 to 1073 K with a gas flowrate of 50 mL min^−1^ in pure nitrogen atmosphere. Five typical heating rates 5, 10, 20, 30 and 40 K min^−1^ were applied. The sample particles were placed into an alumina crucible with a mass of approximately 5 ± 0.2 mg in each test. A flow cell with a recommended temperature of 280 °C was applied to the connection between the TG and FTIR spectrometer to prevent the condensation of gaseous products of the cable sheath. The spectral range was set as 4000–450 cm^−1^ with a scan frequency of eight times.

### 2.4. Calorimeter Measurements

The MCC tests of the samples were carried out using a microscale combustion calorimeter (MCC-2, Govmark in New York, NY, USA) based on ASTM D7309 [30] (Method A). A linear heating rate of 60 K min^−1^ was applied in all tests. The temperature of the combustor was set at 1173 K. The heat release of the PVC sheaths of the new and aged cables was measured using the oxygen consumption principle. The heat release rate (HRR) as a function of time and sample temperature was experimentally analyzed and compared.

In general, the ignition of a cable essentially depends on the properties of the polymeric sheath. To obtain detailed information of the ignition risk of PVC sheathing, the time to ignition (TTI) of new and aged cables was measured based on a bench-scale cone calorimeter by Fire Testing Technology. Typical incident heat fluxes of 25, 35, 50, and 75 kW m^−2^ were selected for the ignition tests. 

## 3. Results and Discussion

### 3.1. Thermogravimetric Analysis

**Figure 2:**
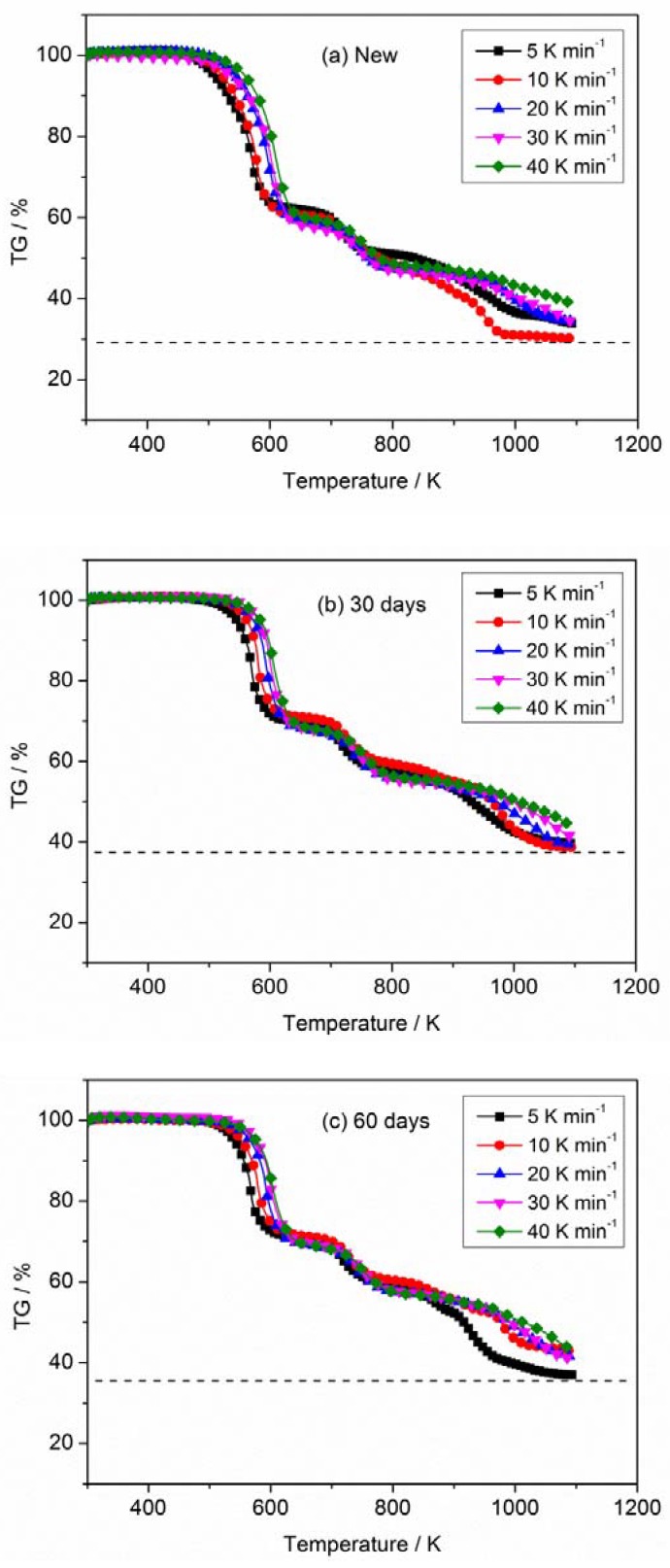
Mass loss (thermogravimetric analysis (TG)) curves of PVC sheaths at different heating rates in nitrogen atmosphere: (**a**) new; (**b**) aged 30 days and (**c**) aged 60 days.

**Figure 3:**
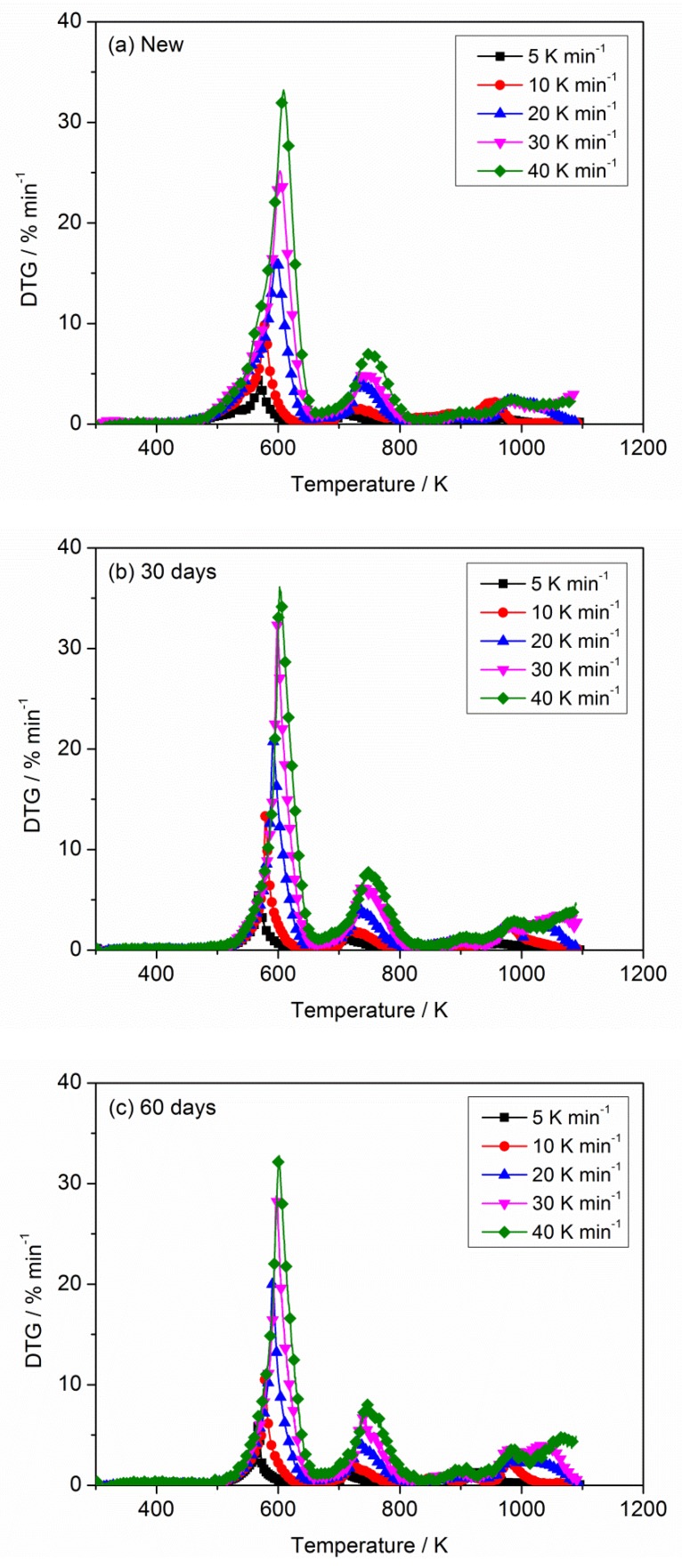
Mass loss rate (derivative thermogravimetric analysis (DTG)) curves of PVC sheath at different heating rates in nitrogen atmosphere: (**a**) new; (**b**) aged 30 days and (**c**) aged 60 days.

Figure 2 and Figure 3 describe the curves of TG and derivative thermogravimetric analysis (DTG) for PVC sheaths of new and aged cables under heating rates of 5, 10, 20, 30 and 40 K min^−1^ in nitrogen atmosphere. It is noted that all TG curves for any sample show similar trends when ignoring the heating rates; the same result is obtained for the DTG curves. The thermal degradation process of the PVC sheath is generally defined as a two-step process, which has been reported and discussed by Zhu et al. [31] and Xie et al. [4]. In the first step, the dehydrochlorination of PVC polymer occurs accompanied by the formation of conjugated double bonds; the second step includes the continuous degradation of PVC polymer with cracking and pyrolysis, resulting in the formation of low hydrocarbons with linear or cyclic structures. Essentially, some weak points, including C–H, C–C, and C–Cl in PVC chains relate to the degradation of PVC polymer [32]. The pyrolysis residue has a conjugated polyene structure with cis- and trans-arrangements [10]. In the present TG and DTG profiles, three significant pyrolysis stages involving two strong peaks and one weak peak can be seen, as shown in Figure 2 and Figure 3, whether in the new or aged cable sheath. The first DTG peak relates to the rapid volatilization and removal of HCl. The cleavage of the conjugated polyene chain and chain structural reconstruction in PVC and additives contribute to the second peak. The third peak may be attributed to the sluggish degradation of remaining residues. In addition, a displacement of TG curves and an increase of DTG peaks with heating rates are observed, as described by previous researchers [32,33,34,35].

**Figure 4:**
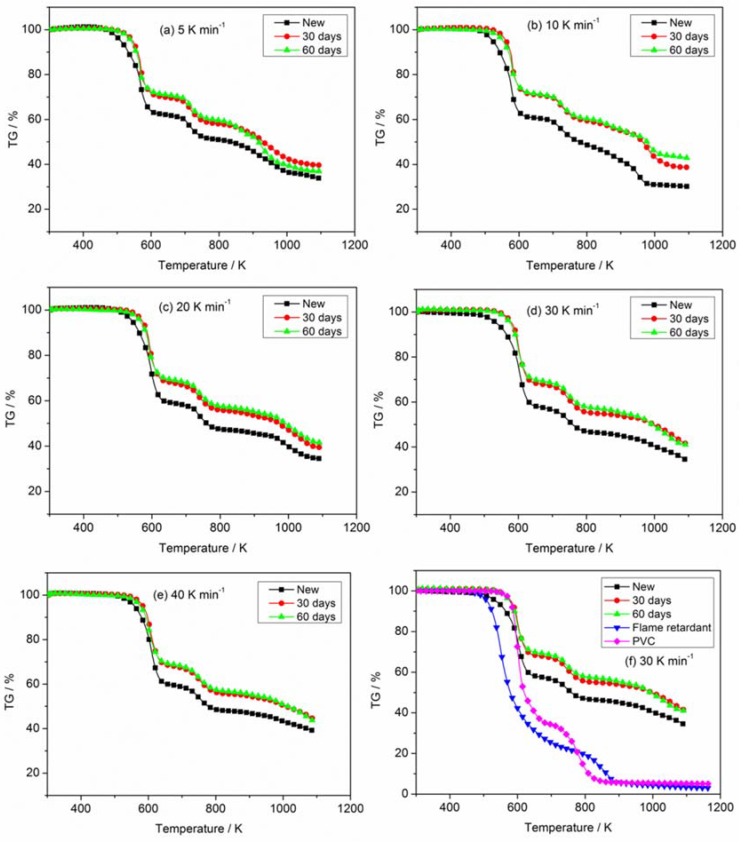
Comparison of TG of PVC sheaths for new and aged cables at different heating rates in nitrogen atmosphere. (**a**) 5 K min^−1^; (**b**) 10 K min^−1^; (**c**) 20 K min^−1^; (**d**) 30 K min^−1^; (**e**) 40 K min^−1^ and (**f**) 30 K min^−1^ involving raw materials.

**Figure 5:**
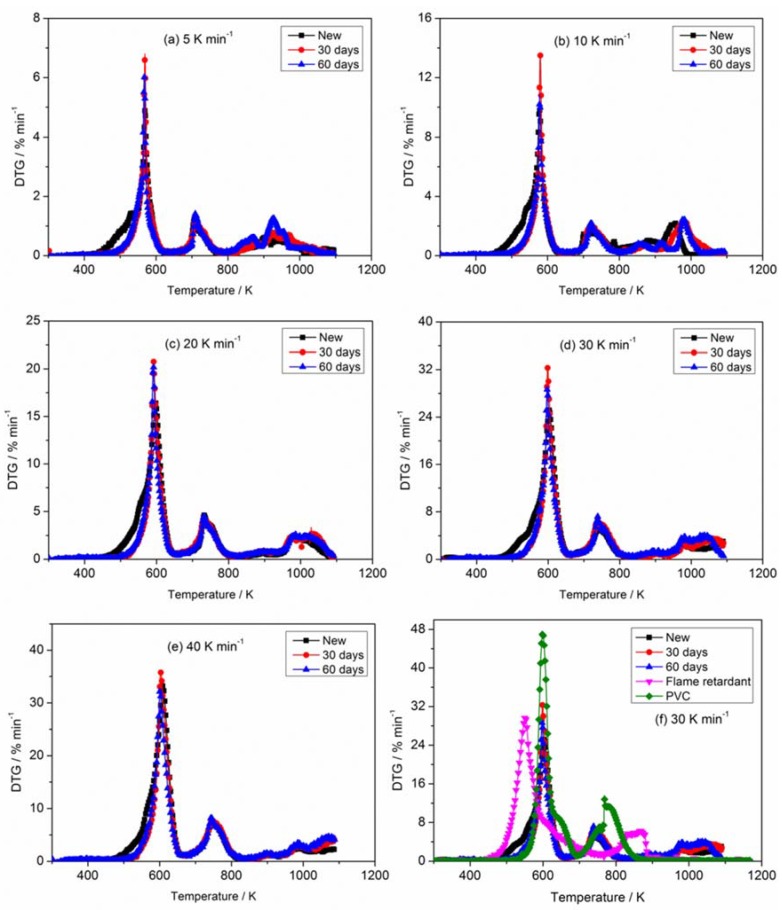
Comparison of DTG of PVC sheaths for new and aged cables at different heating rates in nitrogen atmosphere. (**a**) 5 K min^−1^; (**b**) 10 K min^−1^; (**c**) 20 K min^−1^; (**d**) 30 K min^−1^; (**e**) 40 K min^−1^ and (**f**) 30 K min^−1^ involving raw materials.

The TG and DTG curves corresponding to new and aged PVC sheaths at different heating rates (5, 10, 20, 30 and 40 K min^−1^) in nitrogen atmosphere are depicted in Figure 4 and Figure 5, respectively. Figure 4 illustrates that there is no difference in the TG curves of new and aged PVC sheaths in the initial pyrolysis stage. However, the TG curve of the new cable sheath decreases faster than that of the aged one with increasing sample temperature. The mass of lost residue from the new cable sheath is significantly less than that of the aged one. Namely, the new cable sheath experienced a more sufficient pyrolysis process than the aged one. In addition, the lower the heating rate, the less the residue mass. Overall, the pyrolysis difference between new and aged cable sheaths is clearly visible, regardless of the heating rates. Figure 5 shows that the onset temperature, which is related to the ignition time of polymer in fire, shifts slightly upward for the aged cable sheath at all heating rates. This indicates that the aged cable sheath pyrolyzes later than the new one. The values of DTG peaks for the aged cable sheath are higher than those of the new one. Table 2 lists the typical pyrolysis parameters of PVC sheaths of new and aged cables in nitrogen atmosphere with different heating rates. Note that the changes of chemical composition, structure, and additives in polymer after long-term thermal aging are the main reason for the pyrolysis difference of PVC sheaths between aged and new cables. However, it can be noted that the $T_{onset}$ and $DTG_{peak}$ for the aged sheath of 60 days is lower than that of 30 days, which may be attributed to the complex factors, including the dehydrochlorination, consumption of stabilizers and the aggravated chain scission and cross-linking.

In addition, the effect of the flame-retardant on the thermal degradation of PVC sheath was also analyzed. As shown in Figure 4f and Figure 5f, the $T_{onset}$ of new PVC sheath is lower than that of the pure PVC raw material when the flame retardant is added to the feedstock. However, the new PVC sheath exhibits a lower mass loss rate of while gives a more final residue, as compared with the pure PVC raw material. The whole pyrolysis process has been prolonged after the flame retardant added. Meanwhile, there is an obvious decrease of the main $DTG_{peak}$ for pure PVC raw material. The results are primarily attributed to the formation of volatile SbCl_3_ and SbOCl between the antimony trioxide and PVC during the thermal degradation. Besides, it should be noted the losses of both chlorine and antimony content are clearly seen after thermal aging from the element analysis, which is agreement with the previous works [15,36]. They also proposed that SbCl_3_ production was possible by reaction of HCl with Sb_2_O_3_ without involving the high-temperature disproportionation reactions of intermediate SbOCl. Namely, the higher $T_{onset}$, larger $DTG_{peak}$ and less HCl gas emission may be explained by this point, to some extent. The detailed study of this point is beyond the scope of the current work, taking the experimental design, thermal aging period, etc. into account. However, the relevant research will be conducted in the next plan.

### 3.2. FTIR Analysis

**Figure 6:**
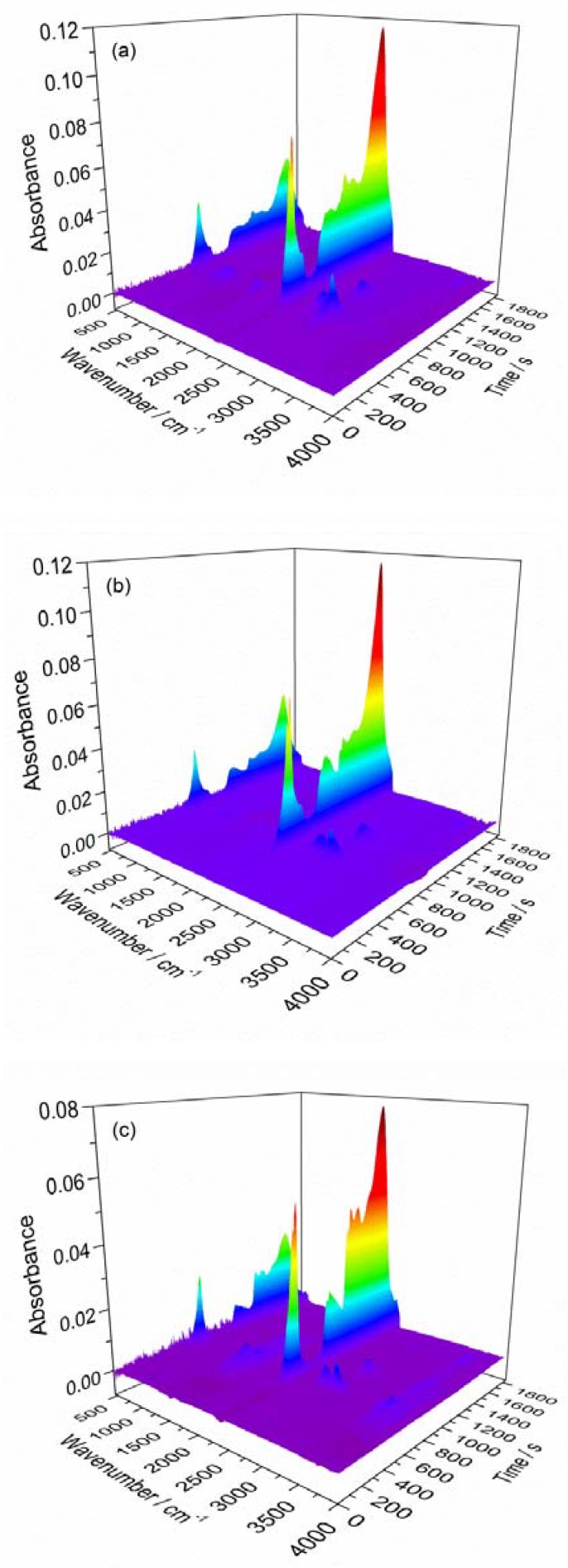
Typical three-dimensional thermogravimetric analysis-Fourier transform infrared (3D TG-FTIR) spectrogram of PVC sheath at a heating rate of 30 K min^−1^: (**a**) new; (**b**) aged 30 days; and, (**c**) aged 60 days.

FTIR spectroscopy is an effective method to analyze the evolved gas products of materials in the pyrolysis process. Figure 6 gives the three-dimensional (3D) surface for the FTIR spectra of gases that are released by new and aged cable sheaths. It is clearly shown that the temperature range of gases released is well consistent with the evolution of DTG, regardless of the third relatively weak peak. In addition, gases are produced from the new cable sheath much earlier than the aged one. However, the kinds and concentrations of gases released in pyrolysis between new and aged cable sheaths have little difference. Table 3 presents typical gases released from PVC sheaths in nitrogen atmosphere at a heating rate of 30 K min^−1^ at different times. It suggests that the main products including HCl, CO_2_, and H_2_O during the whole pyrolysis process are similar for new and aged cable sheaths. However, more dangerous products, such as alkene, benzene, styrene, etc., are not involved in current work. The spectrum band of 2400–2260 cm^−1^ is associated with CO_2_. HCl can be observed in the band range of 3100–2600 cm^−1^, and the absorption band of H_2_O corresponds to 1800–1300 cm^−1^ and 4000–3500 cm^−1^. The spectrum band in the range of 700–550 cm^−1^ is attributed to the stretching vibrations of C–Cl. The results show that the gases from the aged cable sheath are released later, but more quickly, than those by the new one, especially HCl gas.

### 3.3. MCC Analysis

**Figure 7:**
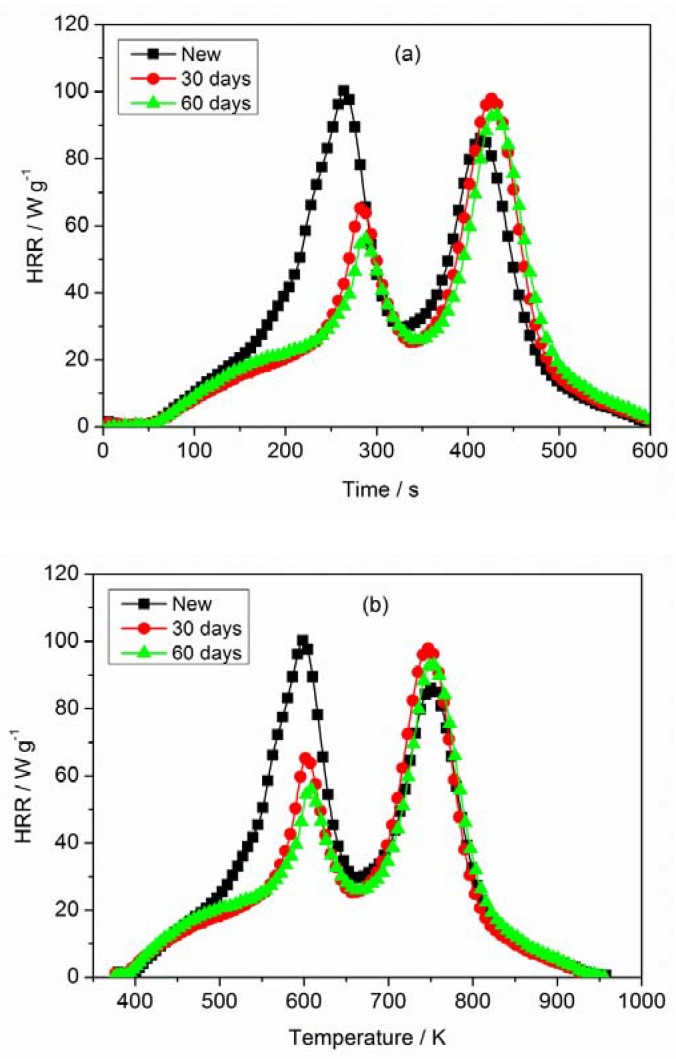
Heat release rate of PVC sheaths for new and aged cables: (**a**) heat release rate (HRR) vs. time and (**b**) HRR vs. temperature.

**Figure 8:**
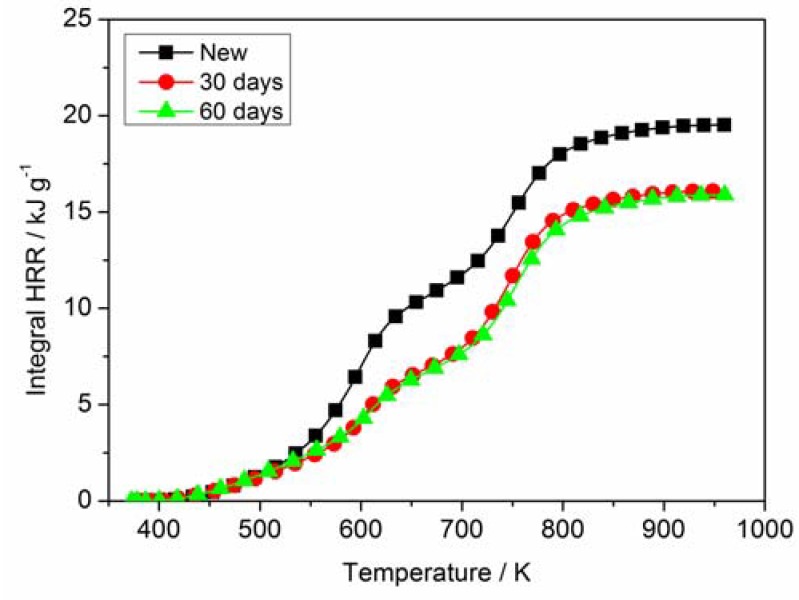
Integral HRR (total heat release (THR)) of PVC sheaths for new and aged cables.

The HRR curves of PVC sheaths for both new and aged cables are shown in Figure 7. Two peaks of heat release rate (PHRRs) can be observed for new and aged sheaths. The value of the first HRR peak for the new cable sheath is greater than that of the aged one, and the value of the second HRR peak has a slight difference. The first HRR peak is produced by the combustion of pyrolysis gases, and the second one is formed by the char oxidation. The result indicates that the faster and more combustible gases were released for aged cable sheath during MCC tests, which can be supported by the measurements of FTIR. The temperature corresponding to two HRR peaks (TPHRRs) laterally shifts upward, and the duration from onset decomposition to PHRR of the aged cable sheath is longer than that of the new one. That means that the decomposition and combustion of aged cable sheath would be slightly weak due to the change of composition and structure. Figure 8 gives the integral HRR (total heat release (THR)) of PVC sheaths for new and aged cables. THR is related not only to the HRR peak, but also to the detailed pyrolysis process. Compared with the aged cable sheath, the THR value of the new cable sheath is relatively high. Table 4 presents the MCC data of PVC sheaths of new and aged cables. Where HRC is the capacity of heat release, PHRR is the maximum value of two peaks, TPHRR is the temperature corresponding to the first peak of HRR, and THR is defined as the value at the final temperature. It implies that the new cable sheath would burn more fiercely and amply, accompanied by more heat release, which is in good agreement with the above TG analysis. 

### 3.4. Cone Calorimetry Analysis

**Figure 9:**
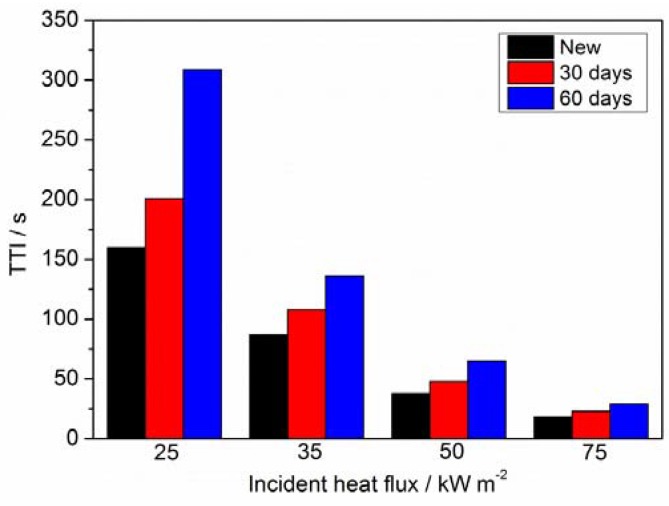
Time to ignition (TTI) of PVC sheaths for new and aged cables with different incident heat fluxes.

Figure 9 shows the time to ignition (TTI) of new and aged cable sheaths under the varied incident heat fluxes ranging from 25 kW m^−2^ to 75 kW m^−2^. The TTI value of the new cable sheath is greater than that of the aged one at different incident heat fluxes. The TTI difference between new and aged cable sheaths decreases with the increasing incident heat flux. The aged cable sheath has a maximal ignition time 93% greater than that of the new cable sheath at 25 kW m^−2^. This result is consistent with the onset temperature and thermal degradation process analysis of pyrolysis. It suggests again that the new cable sheath has a higher ignition risk.

## 4. Conclusions

In the current study, several experiments using TG-FTIR, MCC, and cone calorimetry were employed for PVC sheaths of new and aged cables. The results show that the pyrolysis behavior between aged cable sheaths and new cable sheaths with varied heating rates is markedly different in nitrogen atmosphere. The onset temperature of mass loss for the aged cable sheath is greater than that of the new one, regardless of the heating rates. In addition, the mass of the pyrolysis residue of the aged cable sheath is slightly greater than that of the new cable sheath. This indicates that the new cable sheath starts to pyrolyze more easily and completely than the aged one. It is also concluded that there is a main DTG peak for new and aged cable sheaths under all conditions. The value of the main DTG peak of the aged cable sheath is clearer than that of the new one. The evolved gas that was measured by FTIR spectra illustrates that the aged cable sheath releases pyrolysis gases slightly later but more quickly than the new cable sheath. The results also show that the values of PHRR and THR for the aged cable sheath are clearly less than those of the new one. However, the duration from onset decomposition to PHRR and time to ignition for the aged cable sheath are significantly greater than those of the new cable sheath. It must be noted that the difference of pyrolysis and combustion between the aged sheath with 30 days and aged sheath with 60 days is slight, which may indicate that there is a critical stage during the thermal aging. The pyrolysis and combustion properties of materials change slightly when the materials is aged in a long enough period. Generally, the pyrolysis and combustion properties depend on the material itself under the same condition. In consequence, the modification of chemical composition, chain structure, and additives might be deduced to be the reason for different pyrolysis and combustion properties of the new and aged cable sheaths, eventually resulting in the change of flammability characteristics. Whereas, the currently available evidence is insufficient for this deduction and more research is needed. This work adds to the understanding of the difference in pyrolysis and combustion performances between new and aged cable sheaths. Finally, pyrolysis and combustion of waste plastic allow the obtainment of valuable chemicals, hydrocarbon compounds, combustible, gases, and energy. Knowledge of the pyrolysis mechanisms and combustion properties of typical aged cable sheath will benefit the recycling of plastic waste and energy conversion, which deserves further examination in future study.

## Figures and Tables

**Table 1:** Elemental analysis of new and aged cable sheaths.

Sample	C (%)	H (%)	O (%)	N (%)	Cl (%)	Pb	Sb	Ca
New	33.17	4.16	19.66	0.07	40.67	0.32	1.7	0.25
30 days	30.61	3.63	20.43	0.08	37.38	3.91	1.62	2.34
60 days	28.21	3.24	22.24	0.06	34.61	5.39	1.56	4.69

**Table 2:** Pyrolysis parameters of PVC sheaths for new and aged cables in nitrogen atmosphere with different heating rates.

Sample	Heating Rate (K min^−1^)	T_onset_ (K)	DTG_peak_ (% min^−1^)	Residue_mass_ (%)
New	5	510.41	4.88	33.72
	10	522.36	9.91	30.17
	20	543.28	16.46	34.47
	30	552.96	25.18	34.47
	40	559.12	33.22	39.07
30 days	5	545.84	6.69	39.63
	10	561.77	13.58	38.70
	20	572.44	20.70	39.42
	30	580.89	32.68	41.50
	40	584.08	35.97	44.36
60 days	5	540.75	6.29	36.91
	10	552.54	10.78	42.87
	20	566.09	20.44	41.50
	30	576.14	28.77	41.11
	40	580.24	32.75	43.42

**Table 3:** Gases released from PVC sheaths in nitrogen atmosphere at a heating rate of 30 K min^−1^ with different times.

Sample	Gas	Absorbance			
		8.64 min	10.83 min	16.80 min	30.14 min
New	HCl	7.26 × 10^−3^	1.00 × 10^−2^	4.33 × 10^−3^	1.40 × 10^−3^
	CO_2_	1.07 × 10^−2^	6.49 × 10^−2^	3.59 × 10^−2^	1.81 × 10^−2^
	H_2_O	2.95 × 10^−3^	5.50 × 10^−3^	6.57 × 10^−4^	5.47 × 10^−4^
30 days	HCl	7.17 × 10^−4^	1.70 × 10^−3^	3.59 × 10^−3^	2.96 × 10^−4^
	CO_2_	1.29 × 10^−2^	5.24 × 10^−2^	2.79 × 10^−2^	1.27 × 10^−2^
	H_2_O	1.31 × 10^−5^	1.25 × 10^−3^	7.66 × 10^−5^	1.54 × 10^−5^
60 days	HCl	3.99 × 10^−4^	1.79 × 10^−3^	5.20 × 10^−3^	1.18 × 10^−3^
	CO_2_	1.42 × 10^−2^	5.23 × 10^−2^	2.26 × 10^−2^	7.08 × 10^−3^
	H_2_O	4.71 × 10^−3^	6.35 × 10^−3^	3.74 × 10^−3^	9.39 × 10^−4^

**Table 4:** MCC data of new and aged cable sheaths.

Sample	HRC (J g^−1^ K^−1^)	PHRR (W g^−1^)	TPHRR (°C)	THR (kJ g^−1^)
New	100	100.1	324.2	19.5
30 days	104	98.0	471.4	16.2
60 days	97	93.1	476.8	15.9
